# Undergraduate college students’ awareness and perception of nature - a photovoice study

**DOI:** 10.1186/s12889-023-17455-0

**Published:** 2023-12-15

**Authors:** Whitlee Migl, Haley Mathis, Matthew Spencer, Ruby Hernandez, Jay E. Maddock

**Affiliations:** 1 Department of Environmental and Occupational Health, Texas A&M School of Public Health, College Station, Texas USA; 2 Department of Epidemiology and Biostatistics, Texas A&M School of Public Health, College Station, Texas USA

**Keywords:** Photovoice, Nature, Outdoor, Health, Perception, Knowledge

## Abstract

**Background:**

There has been limited research on college campus’ green spaces and their benefits to students. This study aimed to identify relationships between a Texas campus’s green spaces and students’ knowledge of their health benefits and their perception of their health compared to the campus’ indoor built environments.

**Methods:**

Photovoice was utilized to answer this study’s research questions. Participants were instructed to take a photograph inside a building on campus and one outdoors anywhere on campus. Participants answered a questionnaire containing health-related questions, demographics, and nature relationship questions. Additionally, there was an in-class analysis and discussion to characterize overarching themes, knowledge, and evoked emotions. Frequencies, percentages, and a paired t-test were utilized to investigate the hypothesis that through the application of photovoice, participants would display more knowledge of nature’s health benefits and a better perception of areas providing emotional, mental, physical, and social health benefits when in these green spaces compared to the indoor built environments on campus.

**Results:**

122 students took photographs and answered the questionnaire. 91 students participated in the in-class discussion. Most students felt more positive (80%) and perceived better health with their outdoor location compared to their indoor. They also responded higher to having more positive overall health benefits (63%) outdoors than indoors.

**Conclusions:**

These findings further solidified nature improves overall mood, there is a positive relationship between health and nature, and people are aware of it. Future studies should attempt to identify barriers accessing campus green spaces and develop interventions to encourage students to utilize these spaces.

**Supplementary Information:**

The online version contains supplementary material available at 10.1186/s12889-023-17455-0.

## Introduction

The growth from adolescence to young adulthood involves many emotional, intellectual, mental, physical, and social changes [1]. Transitioning from high school to college is one of the most significant life-altering experiences that people may ever encounter. Many of these issues faced by college students include the continued rising cost of college tuition, work-life balance [2], emotional and mental distress [3], a decrease in leisure satisfaction [4], academic pressures [2–4], and other health-related developmental and behavioral threats [5]. Due to the increased potential of stress related to these areas of possible adversity, there becomes an intensified need for restoration [6].

Ever-increasing pressure resulting from competition to excel academically among university students has led to increased mental fatigue and decreased overall mental health [7]. A previous study of student body leaders in both 2- and 4-year educational institutions reported that mental health was the number one health concern of enrolled students [3]. Due to mental health being influenced by complex biomedical, genetic, and social factors [8], and age also being a significant contributor, it is no surprise that colleges are seeing an increase in students with mental health-related issues [8]. A scoping review on the relationship between nature and the mental health of college students indicated that as little as 10 min of time spent outdoors in nature resulted in a positive effect on mental health [3].

Since at least the early 1980s, it has been found that for prospective students, one of the essential factors in determining what university to attend is the appearance of the campus [9, 10]. For this reason, many college campuses intentionally have nature spaces integrated throughout their grounds, including grass and turf fields, manicured flower beds and trees, gardens, parks, and other natural landscaped areas available for the student body to use [6]. Although the health benefits of nature are not likely a consideration when planning a university’s green spaces, it is a positive byproduct and one of the most influential characteristics for prospective students during campus visits [11]. Over the last few decades, there have been limited studies on the benefits nature has to students’ health, particularly those green spaces already on campuses [9, 12–14]. However, all of these studies indicated either a positive relationship between these campus green spaces and students’ physiological and psychological health [1, 12], their perceived quality of life (QOL) [9], or no significant relationship was identified [13].

Photovoice is a method for conducting research that allows participants to share photographs from their perspective [15]. It is frequently used for uncovering valuable descriptive information that allows for a better understanding and relatability to others [16]. Utilizing photovoice allows people to gain a different perspective of the world from another’s viewpoint they might not have otherwise obtained [17]. Photovoice is a tool designed for participatory research with three primary goals [1] to get individuals to consider and record assets and concerns of their community or self, [2] to encourage discussions of critical issues in the community, or of self, documented in photographs, and [3] to strive for influence amongst policymakers [18]. Three previous college campus studies utilizing photovoice were identified [14, 15, 19, 20]. Findings from these three studies indicated that some of the participants, all of whom were college students, had responses directly related to their perception of nature’s health benefits and the impact nature may have on their QOL [14, 15, 19]. Similarly, this paper aims to identify relationships between a Texas campus’s green spaces and students’ knowledge of the health benefits and the individuals’ perception of their emotional, mental, physical, and social health compared to the campus’ indoor built environments. However, there is a gap in knowledge on what built and natural environment spaces on campus improve mood and overall affect. This study was designed to provide minimal instructions to photo taking to assess which types of photos were taken both indoor and outdoor, how much nature they included and the resulting ratings on mood and affect. It was hypothesized that through the application of photovoice, participants would display more knowledge of nature’s health benefits and a better perception of areas providing emotional, mental, physical, and social health benefits when in these green spaces compared to the indoor built environments on campus.

The three research questions addressed in this study were: (1) Do outdoor photographs elicit more positive emotions and perceived health than indoor photographs? (2) What is the student’s perception of the emotional, mental, physical, and social health of the place where these photographs were taken? (3) What is the participants’ knowledge of the benefits these campus green spaces provide?

## Methods

The methods of this study was adapted from three previously conducted campus photovoice studies [14, 19, 20]. Ethics approval by the university’s International Review Board (IRB#: IRB2022-1561D) was obtained before conducting the study.

### The research site

This study was conducted at a public university campus in Texas with approximately 75,000 students enrolled for the Spring 2023 semester. Approximately five years ago, the campus dedicated approximately 27 acres from its 5,200 acres to develop a garden for extension programs, research, teaching opportunities, and personal use by students and visitors. According to the university’s 2017 Master Plan, a minimum of 439 acres was dedicated to campus green spaces, pocket parks, parks, educational open space, unprogrammed open space, and natural space [21]. Within the last year, an additional estimated 20 acres on the campus was dedicated as an outdoor multipurpose green space for students, faculty, staff, and visitors.

### Participants

Participants were undergraduate public health students enrolled in two sections of a course entitled Public Health Communications during the spring semester of 2023. There were 129 students enrolled between these two sections. The photovoice was one of two choices of assignments provided to students. During the fourth week of classes, the students had the opportunity to decide if they would like to participate in the qualitative photovoice study or an alternate assignment to coincide with the formative research lecture. Of the 129 registered, it was expected that approximately 80, or just over half of the students, would opt to complete the photovoice assignment. However, 125 (96.9%) students elected to participate in the photovoice study. The mean number of participants in other photovoice studies is 21 making this study almost 6 times larger than the average photovoice study [18].

### Procedure

Before starting the study, every student completed a consent form indicating voluntary participation. Those who chose to participate in the photovoice assignment were provided one of two instructions (Appendices A & B) depending on which course section they were registered for. All students were instructed that they had one week to take a single photograph inside one of the buildings on campus and one photograph outdoors anywhere they wanted on the campus grounds. However, one of the sections had more defined instructions to take these photographs in or of places that make them happy. In addition, all students were instructed that no individual may be identifiable in either of their photographs and that it is preferred that no people be photographed at all if possible. The students were free to decide what device they would like to take the photographs with as long as it resulted in digital photographs. They were also free to decide whether to change the photograph’s color or use a filter. After uploading their photographs, the participants completed a survey in Qualtrics consisting of health-related questions, demographics, and general participant and nature relationship questions. Items are described in the measures section below. All photographs were de-identified, and no individual survey responses were shared; they were only included as part of the overall analysis. After completion of the assignment, there was an in-class discussion and Qualtrics survey around the photographs.

The students were given one week to complete and submit all photographs and associated questions in mid-February. This is the beginning of the spring season in Texas with temperate weather. During the in-class discussion day, some of the de-identified photographs were analyzed and discussed as a class in each of the two class sections to characterize overarching themes, knowledge, and evoked emotions. To encourage higher class participation and more accurate individual responses, Poll Everywhere (San Francisco, CA), a real-time audience response system, was utilized with the anonymity feature enabled. Using Poll Everywhere allowed responses to be recorded anonymously by those who wished to participate in the class discussion of the photographs. Analysis and discussion of the photographs in each of the two class sections allowed each participating individual to be validated on their feelings, emotions, and knowledge while allowing other students’ feelings, emotions, and knowledge to be shared and provide a deeper understanding and connection. After presenting and discussing the photographs, both classes were made aware of the purpose of the study. Additional time was set aside for questions and answers (Q&A), feedback, and further discussion.

### Measures

The survey accompanying the 122 participants’ photos included seven demographic questions on age, gender, race/ethnicity, first-generation status, and which section the student was enrolled in. Next, questions about their indoor photograph were assessed. This included rating how they thought the photo’s location affected health, including a breakdown of positive and negative effects on emotional, physical, mental, and social health. They were summed to 4 points for each positive and negative aspect of health they affected. They then rated their feelings while taking photos using items from the Time Spent in Nature (TSN) attitude questions. These questions measure two subscales, positive and negative attitudes, followed by a similar scaling of one through five, with one being strongly disagree and five being strongly agree [22]. These questions were then repeated for the outdoor photograph. The survey ended with questions about how their time is typically spent in nature (Appendix C contains the complete survey).

**Fig. 1:**
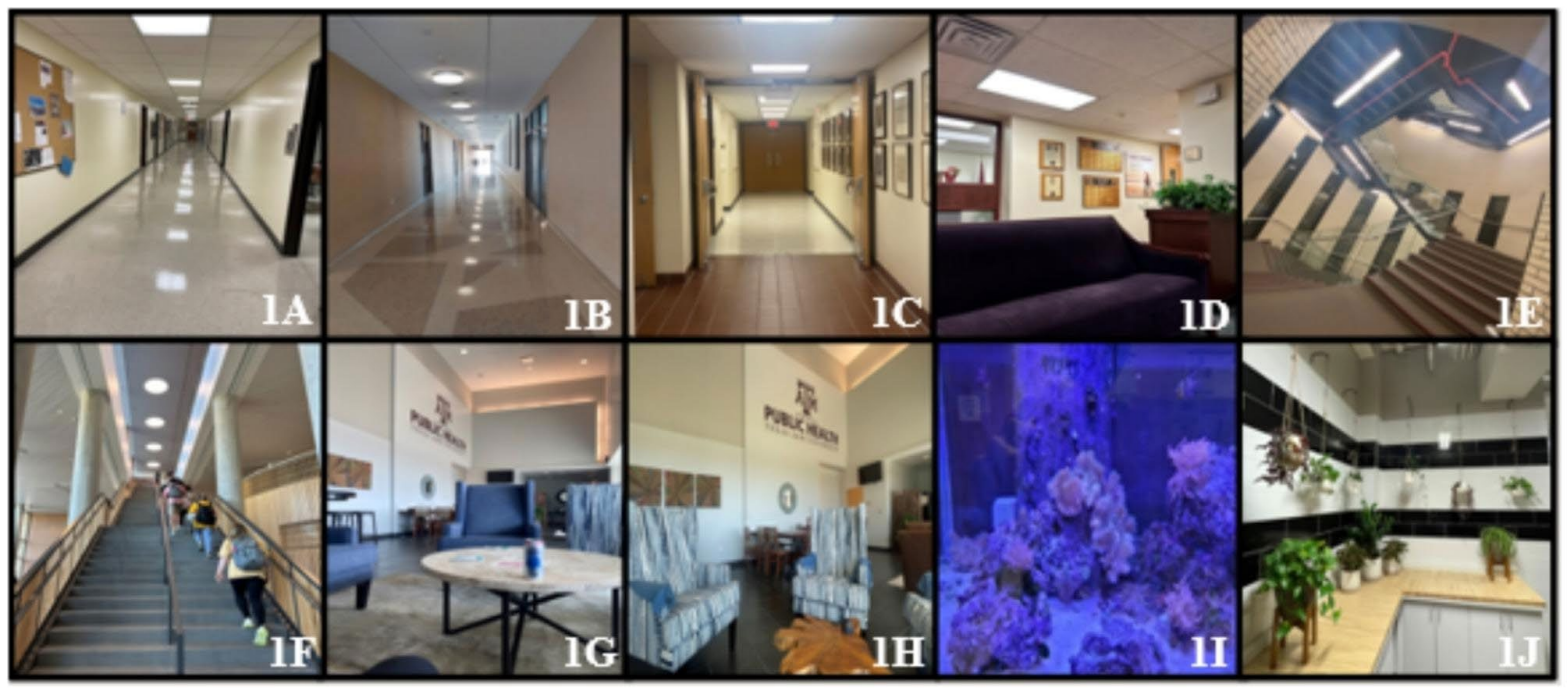
Indoor photographs taken by participants in the photovoice and presented in-class

Additionally, students were asked during the in-class discussion if they had noticed, after seeing ten specific indoor photographs, that all but one contained nature either directly or indirectly (Fig. 1).

**Fig. 2:**
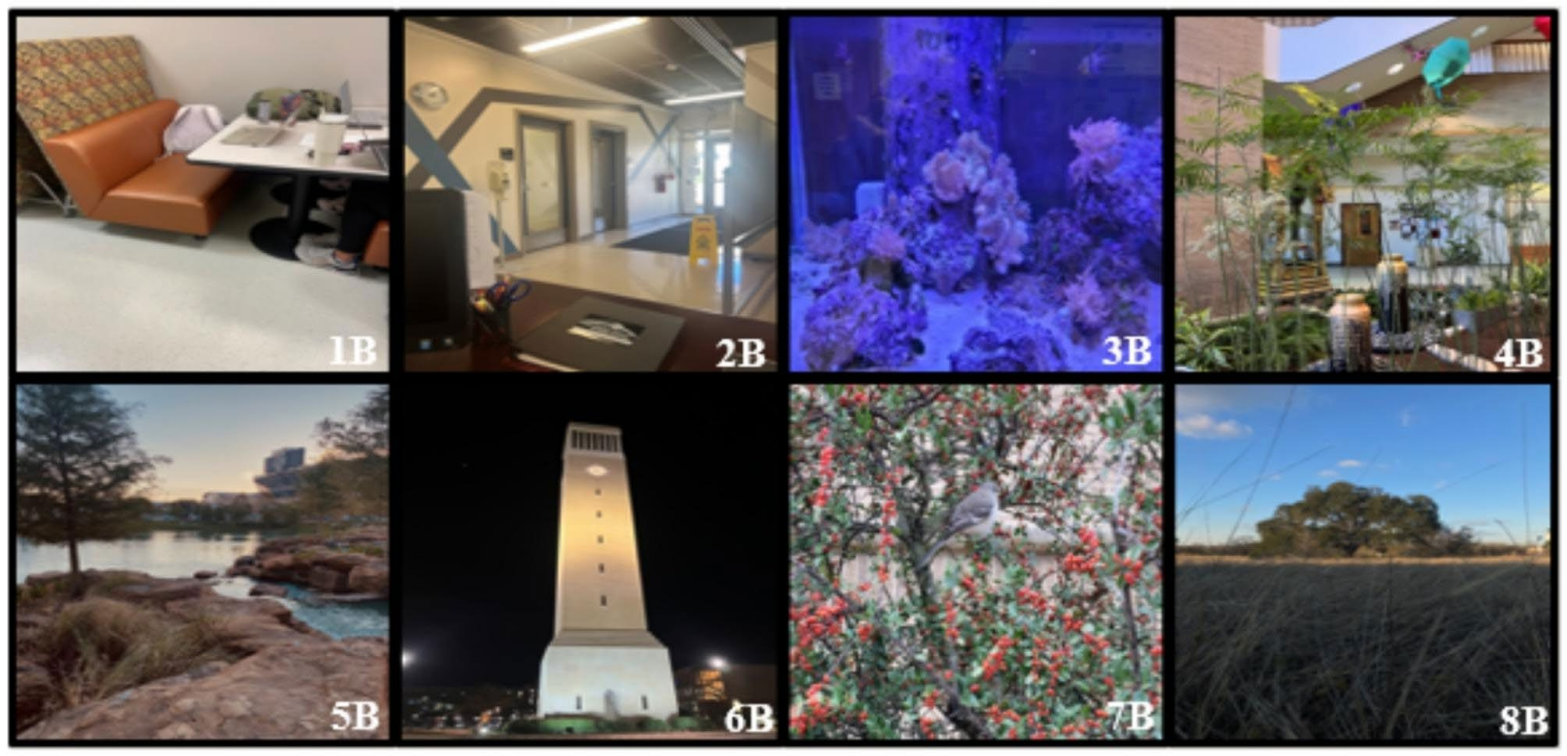
Indoor and outdoor comparison photographs taken by participants in the photovoice and presented in-class

Students were then asked to think about views of nature spaces and light and whether they thought those views affected their mood and mental and physical health. The participants were then shown four comparison photographs of those they had already seen earlier in the lecture. However, this time, it consisted of one of the indoor locations and one of the outdoor locations, and they were asked to decide which of the two made them happier (Fig. 2).

### Data analyses

All individuals’ photographs, responses, and in-class discussions were exported from Qualtrics into two Excel Comma-separated values (CSV) files for coding via Microsoft Office 365 (Microsoft Corporation, Redmond, WA). The CSV files were imported directly into Stata 17 (College Station, TX) for analysis. Summary statistics were calculated on the collected data to summarize the results. Frequencies and percentages were performed to describe any nominal data findings, and a paired t-test was performed to identify any statistical significance between the individual questions when comparing the indoor to outdoor responses. The paired t-test did meet all assumption criteria for use in the analysis, and a p-value of less than 0.05 indicated statistically significant results.

## Results

### Sample characteristics

A total of 122 participants completed the questionnaire, and 91 students participated in the in-class discussion. Almost all participants (99%) were between 18 and 23 years of age. Females (85%) represented most of the participants. The sample was ethnically diverse, with 24% Asian, 4% Black/African American, 60% White, 8% other, and 4% identifying as two or more races. Over a quarter of the participants consider themselves Hispanic or Latino (29%). In addition, just under three-fourths of the participants were from a suburban area (71%), while rural (12%) and urban (16%) combined represented just over one-quarter of the total sample.

### Individual responses to their photographs

The students significantly rated the photos they took outdoors as contributing positively to their health overall. They felt that outdoor locations contributed significantly more to positive health effects and less to negative health effects than indoor locations. The results of the comparison between indoor and outdoor, following the measures outlined above, for the individual perceived benefits and TSN are presented in Table 1.

**Table 1:** Indoor and outdoor photograph questions (N = 122)

Question		Indoor		Outdoor		Paired t-test
		*Mean*	*Standard Deviation*	*Mean*	*Standard Deviation*	*p-value*
Does the location of where the photograph was taken affect health strongly or modestly and positively, negatively, or neither		3.8	0.1	4.2	0.1	**0.0005**
What type of health do you believe this location effects						
	Positive health effects	1.8	0.1	2.5	0.1	**< 0.0001**
	Negative health effects	0.4	0.1	0.1	0.0	**0.0033**

Bolded = p-values < 0.05

### In-class discussions on the photographs

One of the key findings from the in-class discussion was that regardless of the difference in instructions between the two sections, the overall findings showed participants favored photographs with more nature, or those taken outdoors, than those with less nature or indoors, as seen in Table 2.

**Table 2:** In-class responses ratings to photographs

Questions	Responses	*n*	(*%*)
**Indoor Questions Average**			
Which one makes you happier? (*N* = 84)			
	Plant / Natural light or more natural light	41	(48.8)
	No plant / No or little natural light	27	(32.0)
	Indifferent	16	(19.2)
Nature Questions			
Did you notice, except for one photograph from the previous 10, that nine photographs had an element of nature (either direct or indirect)? (*N* = 88)			
	Yes	32	(36.4)
	No	56	(63.6)
When you think of your own preferences inside on campus, do you prefer to be somewhere where you can see outside? (*N* = 88)			
	Yes	84	(95.5)
	No	3	(3.4)
	Indifferent	1	(1.1)
Do you think views of nature spaces and light affect not only your mood but your mental and physical health as well? (*N* = 89)			
	Yes	87	(97.8)
	No	1	(1.1)
	Not sure	1	(1.1)
**Indoor versus Outdoor Questions Average**			
Of the two previously shown photos, which one makes you happier? (*N* = 86)			
	Indoor	15	(18.2)
	Outdoor	65	(74.6)
	Indifferent	6	(7.2)
Did you feel happier taking the indoor or outdoor photo? (*N* = 89)			
	Indoor	11	(12.4)
	Outdoor	66	(74.2)
	Indifferent	12	(13.5)

Of the 88 students who responded to the question, 64% indicated they had not realized there was an element of nature within nine of the ten photographs. The students were then asked when they thought about their preferences when inside on campus, and whether they preferred to be somewhere they could see outside, and 96% of them indicated that they did.

For three of the four comparisons, over 80% chose the outdoor location; in the last comparison, 45% chose the outdoor location, but it was still higher than the indoor location at 41%, which had heavy elements of nature included. Additionally, participants were asked whether they felt happier taking an indoor or outdoor photograph, and 74% indicated they felt happier when taking an outdoor photograph. Lastly, while students preferred indoor photos that contained natural elements, most (63.6%) did not recognize the nature elements in the pictures. The finding that most participants did not realize there were elements of nature in most of the preferred indoor photographs was not surprising but rather intriguing. This further supports that, as humans, we are innately drawn to nature without even knowing we are [23].

## Discussion

It was hypothesized that using photovoice would result in participants intuitively knowing of nature’s health benefits and an increased positive perception of the on-campus green space areas, which provide emotional, mental, physical, and social health benefits compared to the indoor built environments on campus. The hypothesis was significantly supported two-fold by both the significant number of participants at 122 compared to the average of 21 participants; and by all three research questions, (1) Is there a positive difference in emotions and perceived health of the participants’ location where their outdoor photographs were taken compared to their indoor photographs? (2) What is the student’s perception of the emotional, mental, physical, and social health where these photographs were taken? (3) What is the participants’ implicit knowledge of the benefits these campus green spaces provide, as presented by the questionnaires, photographs, and in-class discussions.

Although three previously mentioned university photovoice studies [14, 15, 20] had similar findings that using photovoice for their studies was valuable, this study is both significant and innovative for several reasons. First, it is significant that there were approximately six times the average participants for this study. This innovative study uses photovoice to identify participants’ knowledge and perception of nature and health. It also provides a new approach for future researchers to continue investigating the relationship between health and nature.

The findings of this study further solidified that not only do individuals have a better response to their overall mood while in nature in most instances, but there is also a positive relationship between health and nature, and people are aware of it. Most students did not explicitly notice natural elements in indoor photos that they reported a preference towards. This supports that while people may have an innate desire for nature, they are often unaware. Developing interventions and knowledge-based campaigns may help create this explicit connection between improved mood and time spent in nature. It could help students be more purposive about spending time in nature. The relationship between health and nature has been studied repeatedly in various ways, with similar results that nature positively affects emotions and health [14, 19, 20, 24–26].

### Limitations

While the way this photovoice study was conducted and analyzed is unique to any other photovoice study published, there is still the potential for limitations. The first limitation is that two sections of one course only completed this photovoice study with 122 participants from a college university with over 75,000 students and several hundred different courses available.

Furthermore, because the participants for this study were from a public health course, it is possible they already had prior positive perceptions and vaster knowledge of nature’s benefits to human health. Finally, having the students take one photo outdoors and one indoors may introduce some bias as the areas can differ in terms of functionality and scale.

## Conclusion

Undergraduate students are facing many new life-altering experiences during their transition into college. Some of these experiences result in a negative impact on their health. However, a scoping review on the relationship between nature and the mental health of college students found that even as little as 10 min of their time spent outdoors in nature positively affected their mental health [3]. Therefore, a photovoice study which allows participants to share snapshots from the perspective and emotions of their environment was utilized to identify relationships between a Texas campus’s green spaces and students’ knowledge of their health benefits and their perception of their emotional, mental, physical, and social health when compared to the campus’ indoor built environments.

This study further supports the notion that when individuals are asked to consider and think about nature and its overall impact on mood and health, there is a clear perception and knowledge that nature positively affects both. While exploratory, this study does indicate environments where college students perceive improved mood and overall affect. Given the epidemic of poor mental health on college campuses, future interventions can be tested to increase access to and time spent in these environments. Future studies should include a more diverse sample population, not just two sections from a one-course topic. Other diverse sample populations include comparing other non-public health courses, on-campus organizations, or university-wide. Additionally, future studies should identify barriers students face accessing campus green spaces or develop interventions to encourage students to utilize campus green spaces.

### Electronic supplementary material

Below is the link to the electronic supplementary material.

Supplementary Material 1

Supplementary Material 2

Supplementary Material 3

## Data Availability

Data is available for any reasonable request by the corresponding author.

## Declarations

### Accordance statement

All experiments were performed in accordance with relevant guidelines and regulations.
