# Supplementary material for: Undergraduate college students’ awareness and perception of nature - a photovoice study
Source: BMC Public Health. 2023 Dec 15;23:2515. doi: 10.1186/s12889-023-17455-0 (PMC10722688; doi:10.1186/s12889-023-17455-0)
Supplement: Supplementary file 2 — Supplementary Material 2 [file 12889_2023_17455_MOESM2_ESM.docx]

**Photovoice Study Instructions – Section 501**

1. Take two photographs in/of places that make you happy (one inside of a Texas A&M campus building and one anywhere outdoors on the Texas A&M campus grounds) within the next week.
2. Please do not include any identifiable persons within either of the photographs (it is preferred that no one is in the photograph at all if possible).
3. After taking both photographs, please go to the below Qualtrics link to complete a short questionnaire and to upload your photographs.
4. Please click the link at the end of the questionnaire that will take you to a different survey where you will only input your name to receive a completion grade. It will not be linked with any of your responses from your questionnaire.

https://tamu.qualtrics.com/jfe/form/SV_0oiM4NCDi1uX29o
