# Supplementary material for: Undergraduate college students’ awareness and perception of nature - a photovoice study
Source: BMC Public Health. 2023 Dec 15;23:2515. doi: 10.1186/s12889-023-17455-0 (PMC10722688; doi:10.1186/s12889-023-17455-0)
Supplement: Supplementary file 3 — Supplementary Material 3 [file 12889_2023_17455_MOESM3_ESM.pdf]

Appendix C

Consent Form

Welcome to the research study!

We are interested in determining your personal understanding of your own emotional, mental, physical, and social health, and your knowledge of the benefits nature provides. For this study, you will be presented with information on participating in a photovoice. In addition, you will be asked to answer some questions as you take these photographs. Your responses to the questions will be kept completely confidential.

Each part of the study should take you approximately 15 minutes or less to complete, but you will be given a full week to submit. Your participation in this research is voluntary. You have the right to withdraw at any point during the study. The Principal Investigator of this study can be contacted at Dr. Maddock at maddock@tamu.edu and Whitlee Migl at whitlee@tamu.edu.

By clicking the button below, you acknowledge:

- Your participation in the study is voluntary.
- You are 18 years of age.
- You are aware that you may choose to terminate your participation at any time for any reason and will be allowed to complete a different assignment that will not be utilized in a study.
- You will not be penalized for choosing to complete the alternative assignment and not participate in the study.
- All photographs uploaded to Qualtrics will be de-identified, shared, discussed and potentially utilized for publication. However, the participant is permitted to utilize their own photographs taken for other purposes.

◯ I consent, I wish to participate in the study

◯ I do not consent, I do not wish to participate

Howdy

Howdy,
Thank you for your participation in this study. Please make sure that you have taken both your indoor and outdoor photographs before starting, as they will need to be uploaded during the questionnaire. There will be no repercussions if you decided not to participate in the study at any point during this weeklong study.

Intro

This first section is about your **demographics**

Demographic Questions

How old are you in years?

- ◯ Under 18
- ◯ 18-23
- ◯ 24-30
- ◯ Over 30

What is your gender?

- ◯ Male
- ◯ Female
- ◯ Prefer to self-describe

What is your race?

- ◯ White
- ◯ Black/African American
- ◯ Asian
- ◯ Alaska Native
- ◯ Native Hawaiian
- ◯ American Indian
- ◯ Two or more
- ◯ Other

Do you consider yourself to be Hispanic or Latino?

- ◯ Yes

- No

Are you from a more rural, suburban or urbanized area?

- Rural
- Suburban
- Urban

Are you a first-generation college student?

- Yes
- No

What section are you in?

- 500
- 501

Indoor

The next section is about your **indoor** photograph only

Indoor Photograph Questions

How does the location of where the photograph was taken affect health?

- Strong positive effect on health
- Modest positive effect on health
- Neither positive nor negative effect on health
- Modest negative effect on health
- Strong negative effect on health

If you answered strong or modest effects on health, what type of health do you believe this location effects? If you answered neither positive nor negative effect on health, please select none. (Please select all that apply)

- ☐ Positive emotional health
- ☐ Positive physical health
- ☐ Positive mental health
- ☐ Positive social health
- ☐ Negative emotional health
- ☐ Negative physical health
- ☐ Negative mental health
- ☐ Negative social health
- ☐ None

The following questions will ask about your **feelings** while taking the **indoor** photograph.

While taking the photograph I was in awe?

- ○ Strongly agree
- ○ Somewhat agree
- ○ Neither agree nor disagree
- ○ Somewhat disagree
- ○ Strongly disagree

While taking the photograph I was relaxed?

- ○ Strongly agree
- ○ Somewhat agree
- ○ Neither agree nor disagree
- ○ Somewhat disagree
- ○ Strongly disagree

While taking the photograph I felt energized?

- ○ Strongly agree

- Somewhat agree
- Neither agree nor disagree
- Somewhat disagree
- Strongly disagree

While taking the photograph my mind was clear?

- Strongly agree
- Somewhat agree
- Neither agree nor disagree
- Somewhat disagree
- Strongly disagree

While taking the photograph I forgot my troubles?

- Strongly agree
- Somewhat agree
- Neither agree nor disagree
- Somewhat disagree
- Strongly disagree

While taking the photograph I felt healthier emotionally?

- Strongly agree
- Somewhat agree
- Neither agree nor disagree
- Somewhat disagree
- Strongly disagree

While taking the photograph I felt healthier mentally?

- Strongly agree
- Somewhat agree
- Neither agree nor disagree
- Somewhat disagree
- Strongly disagree

While taking the photograph I felt healthier physically?

- Strongly agree
- Somewhat agree
- Neither agree nor disagree
- Somewhat disagree
- Strongly disagree

While taking the photograph I got excited?

- Strongly agree
- Somewhat agree
- Neither agree nor disagree
- Somewhat disagree
- Strongly disagree

While taking the photograph I felt good about myself?

- Strongly agree
- Somewhat agree
- Neither agree nor disagree
- Somewhat disagree
- Strongly disagree

While taking the photograph I felt good about life?

- Strongly agree
- Somewhat agree
- Neither agree nor disagree
- Somewhat disagree
- Strongly disagree

While taking the photograph I felt bored?

- Strongly agree
- Somewhat agree
- Neither agree nor disagree
- Somewhat disagree
- Strongly disagree

While taking the photograph I felt uncomfortable?

- Strongly agree
- Somewhat agree
- Neither agree nor disagree
- Somewhat disagree
- Strongly disagree

While taking the photograph I felt anxious?

- Strongly agree
- Somewhat agree
- Neither agree nor disagree
- Somewhat disagree
- Strongly disagree

While taking the photograph I felt angry?

- ◯ Strongly agree
- ◯ Somewhat agree
- ◯ Neither agree nor disagree
- ◯ Somewhat disagree
- ◯ Strongly disagree

Did you use a filter when you took the photograph?

- ◯ Yes
- ◯ No

Did you use a filter on the photograph after you took it?

- ◯ Yes
- ◯ No

Please upload your indoor photograph here.

How does this photograph make you feel?

Outdoor

The next section is about your **outdoor** photograph only

Outdoor Photograph Questions

How does the location of where the photograph was taken affect health?

- Strong positive effect on health
- Modest positive effect on health
- Neither positive nor negative effect on health
- Modest negative effect on health
- Strong negative effect on health

If you answered strong or modest effects on health, what type of health do you believe this location effects? If you answered neither positive nor negative effect on health, please select none. (Please select all that apply)

- Positive emotional health
- Positive physical health
- Positive mental health
- Positive social health
- Negative emotional health
- Negative physical health
- Negative mental health
- Negative social health
- None

The following questions will ask about your **feelings** while taking the **outdoor** photograph.

While taking the photograph I was in awe?

- Strongly agree
- Somewhat agree
- Neither agree nor disagree
- Somewhat disagree
- Strongly disagree

While taking the photograph I was relaxed?

- Strongly agree
- Somewhat agree
- Neither agree nor disagree
- Somewhat disagree
- Strongly disagree

While taking the photograph I felt energized?

- Strongly agree
- Somewhat agree
- Neither agree nor disagree
- Somewhat disagree
- Strongly disagree

While taking the photograph my mind was clear?

- Strongly agree
- Somewhat agree
- Neither agree nor disagree
- Somewhat disagree
- Strongly disagree

While taking the photograph I forgot my troubles?

- Strongly agree
- Somewhat agree
- Neither agree nor disagree
- Somewhat disagree
- Strongly disagree

While taking the photograph I felt healthier emotionally?

- Strongly agree
- Somewhat agree
- Neither agree nor disagree
- Somewhat disagree
- Strongly disagree

While taking the photograph I felt healthier mentally?

- Strongly agree
- Somewhat agree
- Neither agree nor disagree
- Somewhat disagree
- Strongly disagree

While taking the photograph I felt healthier physically?

- Strongly agree
- Somewhat agree
- Neither agree nor disagree
- Somewhat disagree
- Strongly disagree

While taking the photograph I got excited?

- Strongly agree
- Somewhat agree
- Neither agree nor disagree
- Somewhat disagree
- Strongly disagree

While taking the photograph I felt good about myself?

- Strongly agree
- Somewhat agree
- Neither agree nor disagree
- Somewhat disagree
- Strongly disagree

While taking the photograph I felt good about life?

- Strongly agree
- Somewhat agree
- Neither agree nor disagree
- Somewhat disagree
- Strongly disagree

While taking the photograph I felt bored?

- Strongly agree
- Somewhat agree
- Neither agree nor disagree
- Somewhat disagree
- Strongly disagree

While taking the photograph I felt uncomfortable?

- Strongly agree
- Somewhat agree
- Neither agree nor disagree
- Somewhat disagree
- Strongly disagree

While taking the photograph I felt anxious?

- Strongly agree
- Somewhat agree
- Neither agree nor disagree
- Somewhat disagree
- Strongly disagree

While taking the photograph I felt angry?

- Strongly agree
- Somewhat agree
- Neither agree nor disagree
- Somewhat disagree
- Strongly disagree

Did you use a filter when you took the photograph?

- Yes
- No

Did you use a filter on the photograph after you took it?

- Yes
- No

Please upload your outdoor photograph here.

How does this photograph make you feel?

General

The last section contains **general questions** about **you** and **nature**

General Nature Questions

Did you spend more time indoors, outdoors, or about the same?

- ◯ Indoors
- ◯ Outdoors
- ◯ About the same

During a typical week, how often do you spend time in nature?

- ◯ Never
- ◯ 30 minutes or less
- ◯ 31 minutes to 1 hour
- ◯ 1-2 hours
- ◯ 2-5 hours
- ◯ 6+ hours

If you answered anything other than never to the previous question, what is the reason you are spending time in nature?

- ☐ Sports
- ☐ Exercise/Workout
- ☐ Hobbies
- ☐ Other health benefits (mental or emotional)

☐ Work

☐ Other
